# CD11b suppresses TLR activation of nonclassical monocytes to reduce primary graft dysfunction after lung transplantation

**DOI:** 10.1172/JCI157262

**Published:** 2022-07-15

**Authors:** Melissa Querrey, Stephen Chiu, Emilia Lecuona, Qiang Wu, Haiying Sun, Megan Anderson, Megan Kelly, Sowmya Ravi, Alexander V. Misharin, Daniel Kreisel, Ankit Bharat, G.R. Scott Budinger

**Affiliations:** 1 Division of Pulmonary and Critical Care Medicine and; 2 Division of Thoracic Surgery, Feinberg School of Medicine, Northwestern University, Chicago, Illinois, USA.; 3 Department of Surgery, Washington University School of Medicine in St. Louis, St. Louis, Missouri, USA.

**Keywords:** Immunology, Transplantation, Integrins, Monocytes

## Abstract

Primary graft dysfunction (PGD) is the leading cause of postoperative mortality in lung transplant recipients and the most important risk factor for development of chronic lung allograft dysfunction. The mechanistic basis for the variability in the incidence and severity of PGD between lung transplant recipients is not known. Using a murine orthotopic vascularized lung transplant model, we found that redundant activation of Toll-like receptors 2 and 4 (TLR2 and -4) on nonclassical monocytes activates MyD88, inducing the release of the neutrophil attractant chemokine CXCL2. Deletion of *Itgam* (encodes CD11b) in nonclassical monocytes enhanced their production of CXCL2 and worsened PGD, while a CD11b agonist, leukadherin-1, administered only to the donor lung prior to lung transplantation, abrogated CXCL2 production and PGD. The damage-associated molecular pattern molecule HMGB1 was increased in peripheral blood samples from patients undergoing lung transplantation after reperfusion and induced CXCL2 production in nonclassical monocytes via TLR4/MyD88. An inhibitor of HMGB1 administered to the donor and recipient prior to lung transplantation attenuated PGD. Our findings suggest that CD11b acts as a molecular brake to prevent neutrophil recruitment by nonclassical monocytes following lung transplantation, revealing an attractive therapeutic target in the donor lung to prevent PGD in lung transplant recipients.

## Graphical abstract

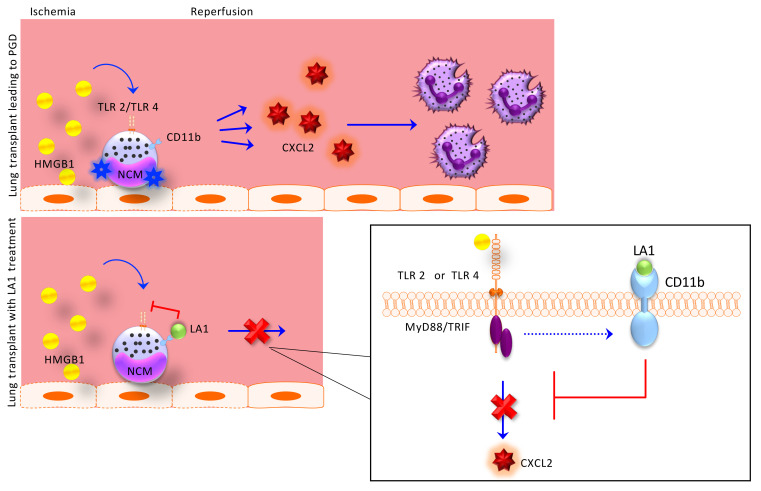

## Introduction

Primary graft dysfunction (PGD) is a syndrome of acute lung injury that develops in 20% to 30% of recipients in the first 72 hours following lung transplantation (1–3). PGD is the predominant risk factor for posttransplant mortality as well as chronic lung allograft dysfunction and contributes to the reduced longevity of lung allografts in comparison with other solid organ transplants (1, 4–13). We reported that the incidence of PGD is significantly higher in patients who undergo lung transplantation for lung fibrosis after COVID-19 compared with patients undergoing lung transplantation for other indications (70% versus 20.8%) (14). Hence, in the absence of strategies to prevent PGD, the increasing number of lung transplantation procedures in patients with COVID-19–induced lung fibrosis is likely to increase the number of lung transplantation procedures complicated by PGD (14). PGD is characterized clinically by arterial hypoxemia accompanied by the presence of patchy alveolar infiltrates on chest radiographs. Histologically, the alveolar spaces show evidence of alveolar endothelial and epithelial injury with neutrophil infiltration and edema fluid within the alveolar space (2, 10). Some clinical features in the donor and recipient have been associated with an increased incidence of PGD; however, the risk attributable to these factors is small and they perform poorly as predictors (15–19). The molecular basis for the variability in the risk and severity of PGD is not known.

In a seminal study, which has since been confirmed by others, Kreisel and colleagues showed that depletion of neutrophils prior to murine lung transplantation attenuated the severity of PGD, causally linking the neutrophilic infiltrates observed in biopsies from patients with PGD to disease pathogenesis (20–23). In murine models of syngeneic and allogeneic transplantation, we found that depletion of donor nonclassical monocytes (NCMs) prior to transplantation reduced the level of the neutrophil-attractive chemokine CXCL2, reduced neutrophil recruitment to the allograft, and ameliorated PGD (24). NCMs are present in the circulation where they are identified as CD14^lo^CD16^+^ in humans and CD11b^+^Ly6C^lo^ in mice. Using flow cytometry and imaging, we found that NCMs are retained in donor lungs adherent to the vascular wall despite clearance of blood from the allograft prior to lung transplantation in mice and humans (24, 25). The mechanisms that induce donor NCMs to produce neutrophil chemoattractants and PGD during lung transplantation remain unknown.

MyD88 is a recruited adaptor protein that is activated by signaling through toll-like receptors (TLRs), a family of pattern recognition receptors. TLRs are known to recognize small molecules originating from pathogens called pathogen-associated molecular patterns (PAMPs), for example lipopolysaccharide (LPS) from the cell wall of gram-negative bacteria. TLRs are also activated by small molecules released from dying cells called damage-associated molecular patterns (DAMPs), for example high-mobility group box B1 (HMGB1), a DNA binding protein normally localized to the nucleus (26–28). During lung transplantation, it has been shown that ischemia induces necroptosis of the endothelium and epithelium, leading to the release of DAMPs (29).

We found that lung transplantation results in the redundant activation of TLR2 and TLR4 in murine NCMs to induce CXCL2 release, neutrophil recruitment, and PGD. We identified an unexpected role for *Itgam*, the gene that encodes CD11b, in modulating CXCL2 production by NCMs in response to TLR signaling. Deletion of *Itgam* in NCMs exacerbated CXCL2 release, neutrophil recruitment, and PGD severity. A pharmacologic agonist of CD11b, leukadherin-1 (LA-1), administered only to the donor lung prior to lung transplantation prevents PGD. The DAMP HMGB1 is increased in human and mouse sera after lung transplantation and activates NCMs to produce CXCL2 via TLR4. Inhibition of HMGB1 in both the donor and recipient prior to lung transplantation attenuates PGD severity. Our studies suggest CD11b and other signaling molecules necessary to activate NCMs might be targeted to the donor lung to reduce the incidence and severity of PGD after lung transplantation.

## Results

### CD11b modulates TLR2 and TLR4 signaling in NCMs.

We sought to develop an in vitro system to explore the upstream signaling events that trigger the release of CXCL2 from primary murine splenic NCMs. Our initial protocol to isolate NCMs began with a cell enrichment step using columns containing beads decorated with antibodies targeting CD11b. NCMs isolated using this protocol showed variation in CXCL2 release in the absence of additional stimulus (Supplemental Figure 1A; supplemental material available online with this article; https://doi.org/10.1172/JCI157262DS1). CD11b is a surface integrin that can promote cell migration through reciprocal binding to cognate antigens such as ICAM-1 (30–32). While other roles for CD11b have not been described in monocytes, CD11b has been reported to negatively regulate NF-κB and interferon signaling in human and murine macrophage populations in response to TLR ligation (33–35). We therefore wondered whether antibodies in the column might inactivate CD11b during NCM isolation to enhance TLR signaling. Accordingly, we developed an alternative protocol to isolate NCMs via a process of negative selection using anti-CD3ε and anti-CD19 microbeads with flow cytometry that did not include anti-CD11b antibodies or microbeads (Supplemental Figure 1, B and C, and Supplemental Table 1). Using this protocol, we saw little induction of CXCL2 release in unstimulated NCMs and reduced levels of CXCL2 response to TLR agonists (Supplemental Figure 1A).

**Figure 1:**
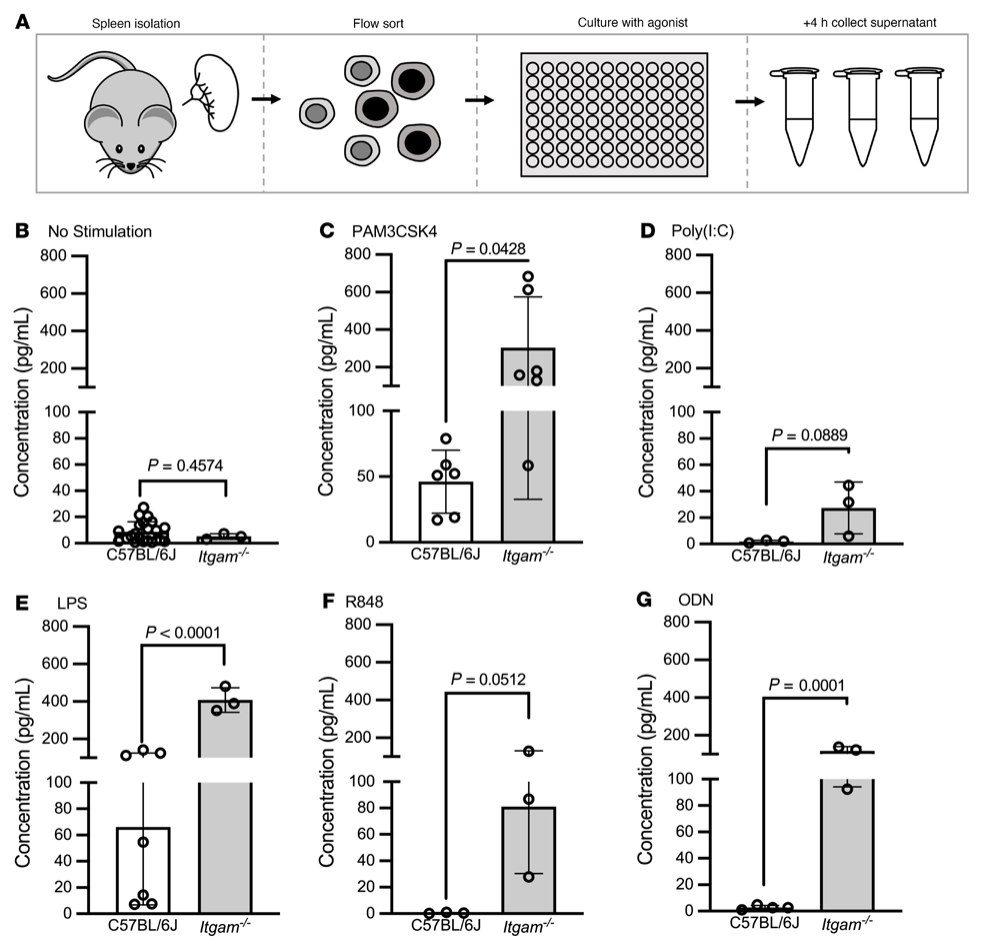
*Itgam^–/–^* increases CXCL2 production in response to TLR2 and TLR4 agonists. (**A**) Splenic NCMs were flow cytometry sorted from wild-type (C57BL/6J) and *Itgam^–/–^* mice and stimulated with TLR agonists as indicated for 4 hours, after which the supernatants were collected for ELISA analysis. (**B**–**G**) PAM3CSK4, a TLR2 agonist; poly(I:C), a TLR3 agonist; lipopolysaccharide (LPS), a TLR4 agonist; resiquimod (R848), a TLR 7/8 agonist; and oligodeoxynucleotide (ODN), a TLR9 agonist were administered at a dose of 1 μg. Each symbol represents 50,000 NCMs, and approximately 100,000–200,000 NCMs were isolated from an individual mouse. *P* values above the horizontal lines were calculated using a 2-tailed, unpaired *t* test.

**Figure 2:**
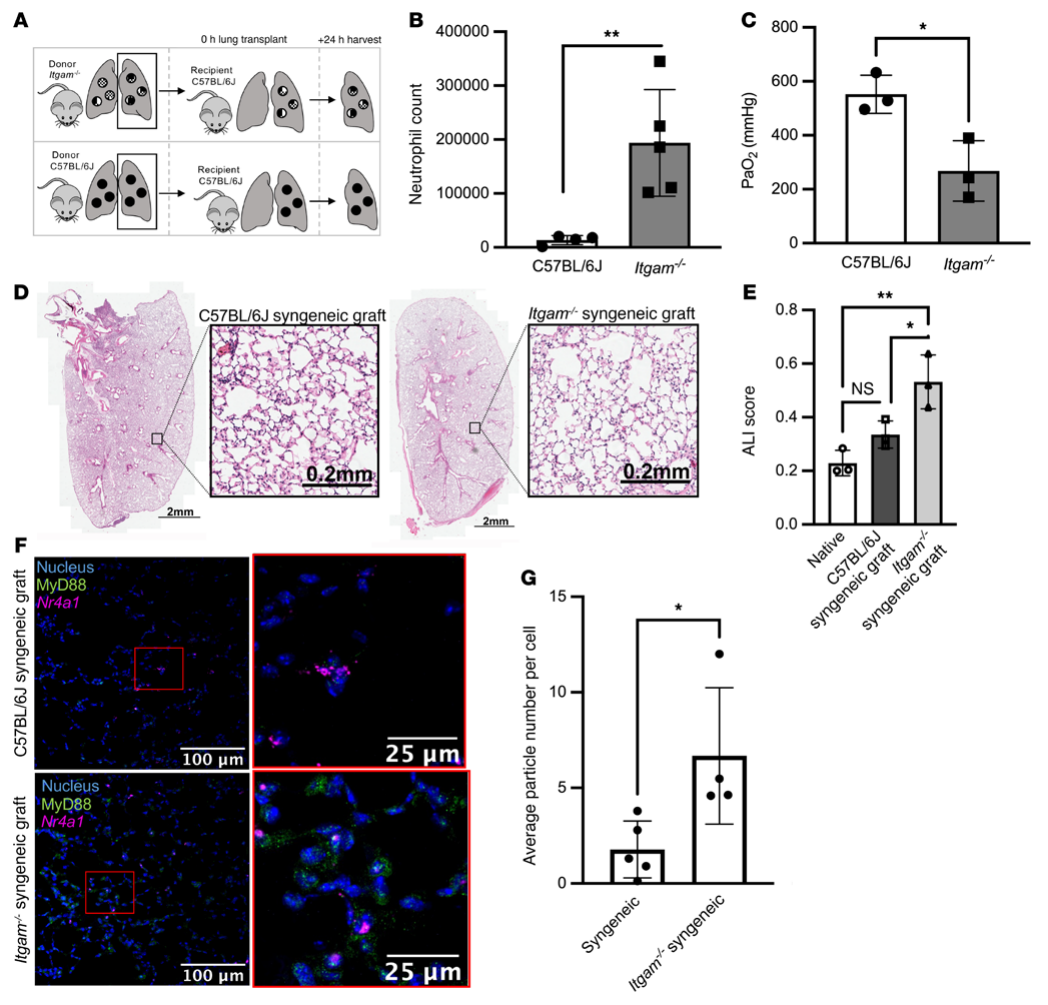
*Itgam^–/–^* donor lungs exacerbate development of PGD. (**A**) Schematic for murine syngeneic lung transplants. (**B**) Lung allografts were harvested 24 hours after lung transplantation and neutrophil numbers were measured using flow cytometry. **P = 0.0019 by 2-tailed, unpaired *t* test. (**C**) Arterial blood was obtained for blood gas analysis while the mouse was receiving 100% oxygen via mechanical ventilation immediately prior to harvest. *P = 0.0206 by 2-tailed, unpaired *t* test. (**D**) Representative H&E-stained allografts from wild-type and *Itgam^–/–^* mice. (**E**) Acute lung injury (ALI) scores based on histologic evaluation. *P = 0.0329, **P = 0.0046 by 1-way ANOVA with Dunnett’s test to correct for multiple comparisons. NS, not significant. (**F**) Lung sections from allografts of wild-type and *Itgam^–/–^* mice were stained using a combination of single-molecule fluorescence in situ hybridization (RNAScope) and immunohistochemistry (blue: nuclear stain, green: MyD88, magenta: *Nr4a1*). Original magnification, ×400. (**G**) MyD88-containing aggregates per *Nr4a1*-positive NCM in C57BL/6J and *Itgam^–/–^* syngeneic grafts. *P = 0.0261 by 2-tailed, unpaired *t* test. Each symbol represents an individual mouse.

We then used this system to explore the role of CD11b in TLR signaling in NCMs. We isolated NCMs from the spleens of C57BL/6J wild-type and *Itgam^–/–^* mice. At baseline, *Itgam^–/–^* mice had lung and spleen phenotypes similar to those of C57BL/6J (Supplemental Figure 2, A–D). NCMs from *Itgam^–/–^* mice showed enhanced CXCL2 release in response to TLR2, TLR4, and TLR9 agonists (Figure 1, A–G). We observed similar enhancements in the release of other inflammatory cytokines in NCMs from *Itgam^–/–^* mice in comparison with those from wild-type mice (Supplemental Figure 3). We have shown that CXCL2 released from donor-derived NCMs retained in the allograft after reperfusion are necessary for the recruitment of neutrophils and development of PGD after allogeneic lung transplantation in mice, a finding that has since been confirmed by others in a murine model of ischemia reperfusion (21, 36). Accordingly, we performed syngeneic lung transplantations using *Itgam^–/–^* mice (C57BL/6J background) as donors and wild-type C57BL/6J mice as recipients (Figure 2A). We chose syngeneic transplantation, as the severity of PGD is mild in this model compared with allogeneic transplantation and the confounding effects of innate alloimmunity are not present (24). Compared with wild-type to wild-type lung transplants, lung transplants from *Itgam^–/–^* donor lungs showed significantly increased neutrophil infiltration and a lower PaO_2_/F_I_O_2_ indicative of worsened PGD (Figure 2, B and C). As expected, syngeneic transplantation resulted in minimal evidence of histologic injury in wild-type to wild-type animals, but resulted in increased neutrophil recruitment and a worse lung injury score in *Itgam^–/–^* into wild-type animals (Figure 2, D and E). MyD88 is normally present in the cytoplasm as a homodimer that forms tiny punctae uniformly distributed throughout the cytoplasm. Upon TLR stimulation, large (>1 μm) multiprotein aggregates containing MyD88 form in the cytoplasm (37). We reasoned that detection of these aggregates in NCMs in tissue sections from the allografts after lung transplantation could be used to measure the activation of MyD88 in vivo. Accordingly, we used single-molecule fluorescent in situ hybridization (smFISH) (RNAScope) to identify cells expressing *Nr4a1*, which encodes a transcription factor whose expression is confined to NCMs within the distal lung. We combined this with immunofluorescence staining using an antibody against MyD88 and quantified the number of MyD88-positive aggregates in *Nr4a1*-positive NCMs (Figure 2F). The number of MyD88-containing aggregates increased in NCMs from *Itgam^–/–^* syngeneic grafts compared with those from wild-type syngeneic transplants (Figure 2G).

### Redundant signaling through TLR2 or TLR4 in donor NCMs is necessary for CXCL2 release and PGD.

**Figure 3:**
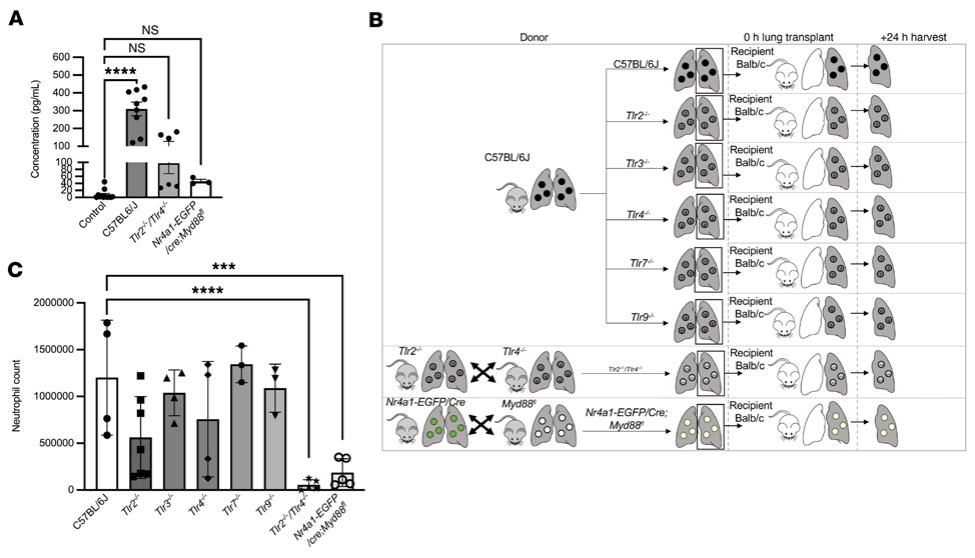
Activation of TLR2 or TLR4 is necessary for CXCL2 release from NCMs and the development of PGD after murine lung transplantation. (**A**) CXCL2 in supernatants from flow cytometry–sorted splenic NCMs from C57BL/6J, *Tlr2^–/–^* *Tlr4^–/–^*, and *Nr4a1-EGFP/cre* *Myd88^fl/fl^* mice after stimulation with lysed endothelial supernatant for 4 hours. ****P < 0.0001 by 1-way ANOVA with Dunnett’s correction for multiple comparisons. (**B**) Schematic for the allogeneic transplantations in **C**. Donor mice were C57BL/6J (wild type), *Tlr2^–/–^*, *Tlr3^–/–^*, *Tlr4^–/–^*, *Tlr7^–/–^*, *Tlr9^–/–^*, *Tlr2^–/–^* *Tlr4^–/–^*, and *Nr4a1-EGFP/cre* *Myd88^fl/fl^*. All recipients were BALB/c mice. Lungs were harvested 24 hours after transplantation. (**C**) Neutrophils were quantified by flow cytometry analysis of lung homogenates. ***P = 0.0006, ****P < 0.0001 by 1-way ANOVA with Dunnett’s multiple comparison test, as detailed in Supplemental Figure 5. NS, not significant. Each symbol represents an individual mouse.

**Figure 4:**
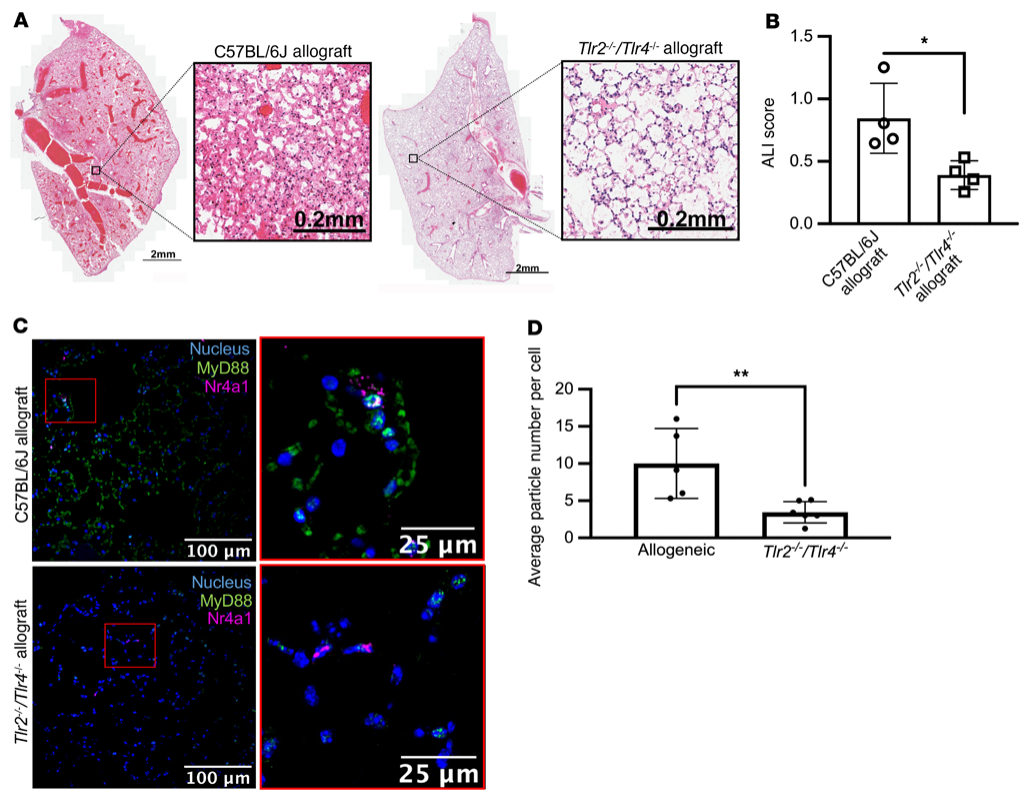
Activation of TLR2 or TLR4 is necessary for the development of PGD after murine lung transplantation. (**A**) Representative H&E staining of wild-type and *Tlr2^–/–^* *Tlr4^–/–^* allografts show less necrosis and inflammatory infiltrate with preservation of lung structure. (**B**) Acute lung injury (ALI) scores based on histologic evaluation. *P = 0.0162 by 1-way ANOVA with Dunnett’s multiple comparisons test, as detailed in Supplemental Figure 5. (**C**) Lung sections from wild-type and *Tlr2^–/–^* *Tlr4^–/–^* mice were analyzed using a combination of RNAScope and immunohistochemical staining of allografts (blue: nuclear stain, green: MyD88, magenta: *Nr4a1*). Original magnification, ×400. (**D**) MyD88-containing aggregates per *Nr4a1*-positive NCM in C57BL/6J and *Tlr2^–/–^* *Tlr4^–/–^* allografts. **P = 0.0032 by 1-way ANOVA with Dunnett’s multiple comparisons test, as detailed in Supplemental Figure 5. Each symbol represents an individual mouse. The controls for ALI score and average particle number per cell are identical to those in Figures 6 and 8.

Having established a model to flow sort NCMs without engaging CD11b, we examined the specific upstream signaling events that activate MyD88-induced CXCL2 release from donor lung NCMs during lung transplantation. MyD88 is known to be activated in response to signaling through TLRs (38, 39). Our studies in isolated splenic NCMs implicated TLR2 and TLR4 signaling in the release of CXCL2 (Figure 1B). We sought to determine whether redundant activation of TLR2 and TLR4 in NCMs is necessary for PGD after lung transplantation. NCMs isolated from spleens of *Tlr2^–/–^* *Tlr4^–/–^* and *Nr4a1-EGFP/cre* *Myd88^fl/fl^* mice had a significantly lower production of CXCL2 than wild-type NCMs when stimulated with lysed endothelial supernatant (LES), a proxy for necroptotic cells during lung transplantation (Figure 3A). We then performed allogeneic transplants using *Tlr2^–/–^*, *Tlr3^–/–^*, *Tlr4^–/–^*, *Tlr7^–/–^*, *Tlr9^–/–^*, *Tlr2^–/–^* *Tlr4^–/–^*, and *Nr4a1-EGFP/cre* *Myd88^fl/fl^* donors (C57BL/6J background) into BALB/c recipients (Figure 3B). When allogeneic lung transplantation was performed using single-TLR-knockout donors, neutrophil influx into the graft 24 hours after transplantation was similar to wild-type donors. Mice doubly deficient in *Tlr2* and *Tlr4* had reduced neutrophils, as did *Nr4a1-EGFP/cre* *Myd88^fl/fl^* donors (Figure 3C and Figure 4, A and B). Consistent with these findings, the number of MyD88-containing aggregates in NCMs was reduced in lung allografts from *Tlr2^–/–^* *Tlr4^–/–^* donors in comparison with wild-type donors (Figure 4, C and D).

### HMGB1 contributes to NCM activation and PGD after lung transplantation.

**Figure 5:**
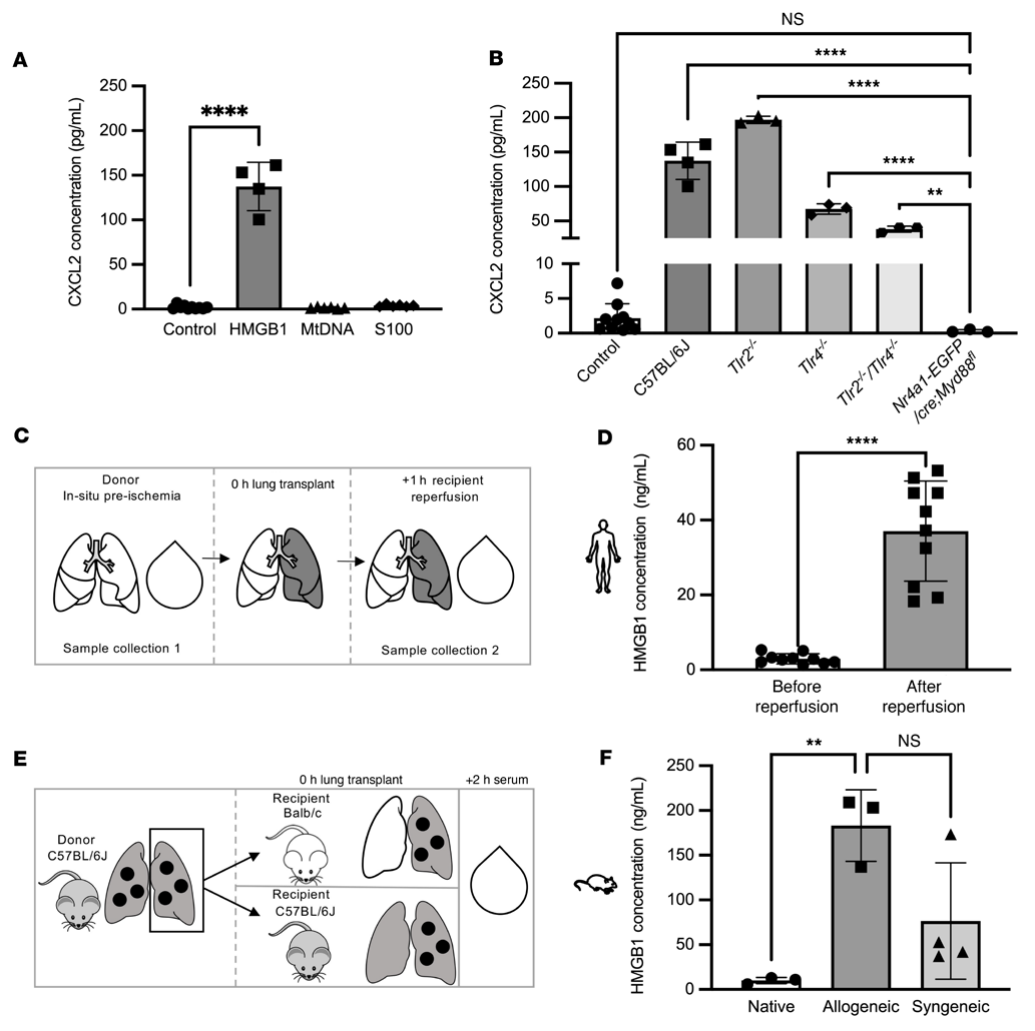
HMGB1 activates TLR2 and TLR4 to induce the expression of CXCL2 in NCMs. (**A**) Splenic NCMs were flow cytometry sorted from wild-type (C57BL/6J) mice and stimulated with putative DAMPs as indicated for 4 hours, after which the supernatants were collected for ELISA analysis. ****P < 0.0001 by 1-way ANOVA with Tukey’s multiple comparisons test. Mitochondrial DNA (mtDNA), S100 proteins (S100), and HMGB1 were administered at a dose of 1 μg. (**B**) Splenic NCMs were flow cytometry sorted from wild-type (C57BL/6J), *Tlr2^–/–^*, *Tlr4^–/–^*, and *Tlr2^–/–^* *Tlr4^–/–^* mice and treated with HMGB1 (1 μg), and CXCL2 was measured in the supernatant 4 hours later. **P = 0.0022, ****P < 0.0001 by 1-way ANOVA with Tukey’s multiple comparisons test. NS, not significant. (**C**) Schematic for human sera collection in **D**. (**D**) Blood samples from patients undergoing lung transplantation were obtained immediately prior to and 24 hours after reperfusion and analyzed for HMGB1. ****P < 0.0001 by 2-tailed, paired *t* test. (**E**) Schematic for murine sera collection. (**F**) HMGB1 serum concentrations in native, allogeneic, and syngeneic lung transplants. **P = 0.0073 by 1-way ANOVA with Dunnett’s correction for multiple comparisons. In **A** and **B**, each symbol represents 50,000 NCMs, and approximately 100,000 to 200,000 NCMs were isolated from an individual mouse; each symbol in **D** and **F** represents an individual mouse or human.

DAMPs are a group of proteins normally localized to the intracellular space that are released upon cell necrosis. In the extracellular space, these proteins signal through pattern recognition receptors, including TLRs, to activate inflammatory pathways. We flow cytometry sorted splenic NCMs from wild-type mice and stimulated them with a panel of DAMPs, including recombinant HMGB1, mitochondrial DNA, and S100 proteins. HMGB1 significantly increased CXCL2 production in NCMs, whereas the other DAMPs did not (Figure 5A). HMGB1 is a ubiquitous DNA binding protein that is normally localized to the nucleus and is a known agonist of both TLR2 and TLR4 (40–42). Accordingly, we flow cytometry sorted NCMs from wild-type, *Tlr2^–/–^*, *Tlr4^–/–^*, and *Tlr2^–/–^* *Tlr4^–/–^* mice and measured CXCL2 concentrations in the media after stimulation with HMGB1. Compared with stimulated NCMs from wild-type mice, CXCL2 concentrations in the media were lower in stimulated NCMs from *Tlr4^–/–^* mice and mice doubly deficient in *Tlr2* and *Tlr4*, with levels similar to those of unstimulated wild-type NCMs (Figure 5B). Surprisingly, we did not observe attenuation of HMGB1 signaling in NCMs from *Tlr2^–/–^* mice. We then measured serum HMGB1 levels in peripheral blood from patients collected 24 hours after reperfusion of the allograft and compared them to levels collected before transplantation. HMGB1 levels in human recipients were significantly increased after reperfusion in comparison with before transplant (Figure 5, C and D). Consistently, serum HMGB1 levels in mice 2 hours after allogeneic transplantation were significantly higher than those in naive mice (Figure 5, E and F).

### A small molecule inhibitor of HMGB1 reduces PGD severity after lung transplantation.

**Figure 6:**
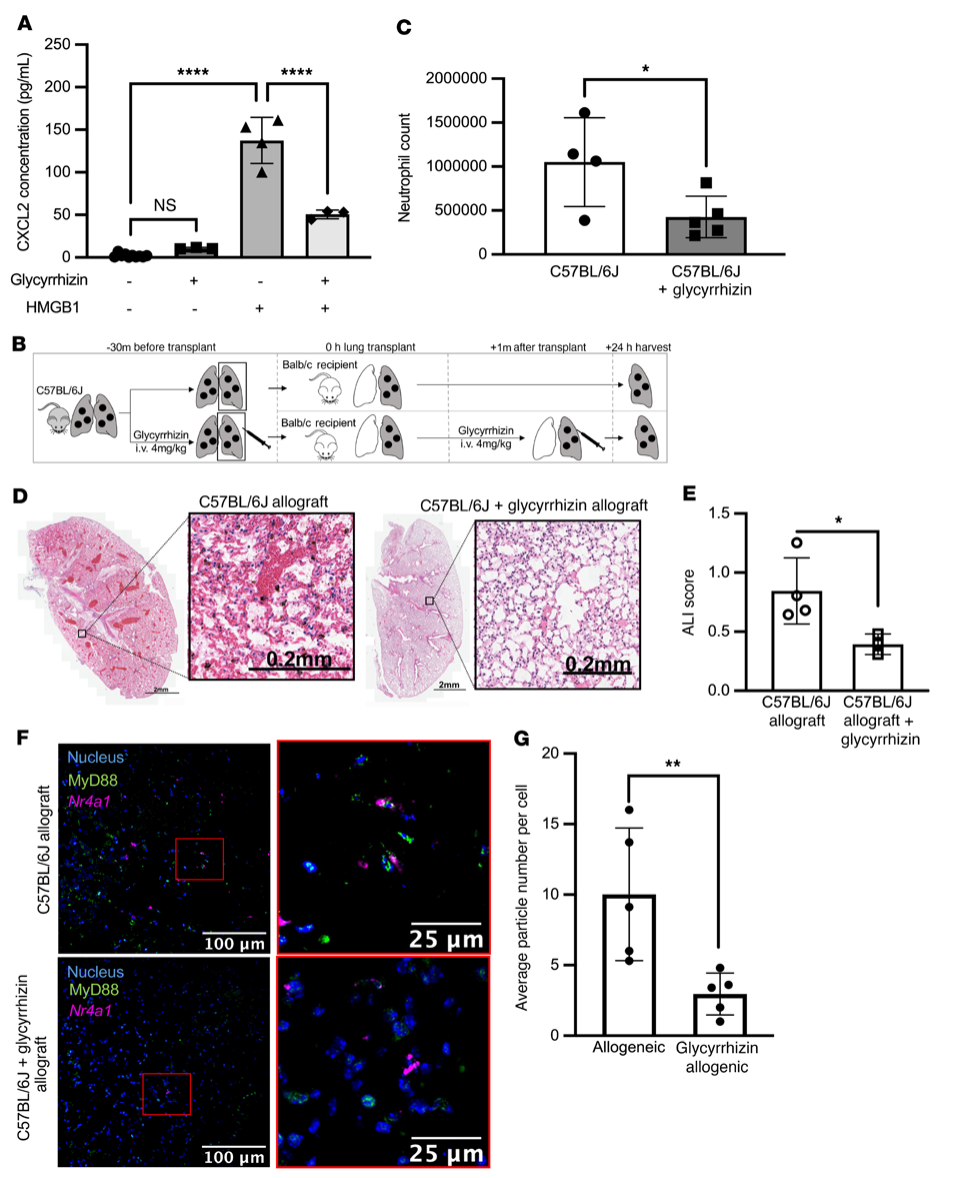
Glycyrrhizin inhibits HMGB1 activation of NCMs and prevents PGD in mice. (**A**) Splenic NCMs were flow cytometry sorted from wild-type (C57BL/6J) mice and treated with glycyrrhizin (20 μM) followed 20 minutes later by HMGB1 (10 μg/mL), and CXCL2 concentrations in the supernatants were measured 4 hours later. ****P < 0.0001 by 1-way ANOVA with Tukey’s multiple comparisons test. (**B**) Schematic for the allogeneic transplantations in **C**–**E**. Donor mice were C57BL/6J and recipients were BALB/c. Glycyrrhizin was administered at 4 mg/kg intravenously to the donor 30 minutes before harvest and to the recipient immediately after reperfusion. (**C**) Lung allografts were harvested 24 hours after lung transplantation and neutrophil numbers were quantified by flow cytometry analysis of lung homogenates. *P = 0.0235 by 1-way ANOVA with Dunnett’s correction for multiple comparisons, as detailed in Supplemental Figure 5. Each symbol represents an individual mouse. (**D**) Representative H&E staining of allografts with and without glycyrrhizin treatment. (**E**) Acute lung injury (ALI) scores for the images in **D**. *P = 0.0259 by 1-way ANOVA with Dunnett’s correction for multiple comparisons (see Supplemental Figure 5). (**F**) Lung sections from allografts after syngeneic lung transplantation with or without treatment with glycyrrhizin using RNAScope and immunohistochemical staining (blue: nuclear stain, green: MyD88, magenta: *Nr4a1*). Original magnification, ×400. (**G**) MyD88 particle counts per *Nr4a1*-positive NCM in C57BL/6J allografts with and without glycyrrhizin treatment. **P = 0.0025 by 1-way ANOVA with Dunnett’s multiple comparisons test, as detailed in Supplemental Figure 5. Each symbol represents 50,000 NCMs. The controls for ALI score and average particle number per cell are identical to those in Figures 4 and 8.

Glycyrrhizin binds directly to HMGB1 and limits its inflammatory effects in other models of inflammation (43–45). We pretreated flow cytometry–sorted splenic NCMs from wild-type mice with glycyrrhizin, which prevented the increase in CXCL2 in the media in response to HMGB1 (Figure 6A). To model the time course of lung transplantation in humans, we then pretreated donor mice with intravenous glycyrrhizin 2 hours prior to allogeneic lung transplantation and gave intravenous glycyrrhizin to the recipient immediately after reperfusion (Figure 6B). Compared with untreated mice, mice treated with glycyrrhizin showed reduced neutrophil recruitment to the lung allograft and reduced PGD severity (Figure 6, C–E). Consistent with these findings, the number of MyD88-containing aggregates in NCMs was reduced in lung allografts after treatment of the donor and recipient with glycyrrhizin (Figure 6, F and G).

### Treatment of the donor lung with a CD11b agonist, LA-1, attenuates PGD severity.

**Figure 7:**
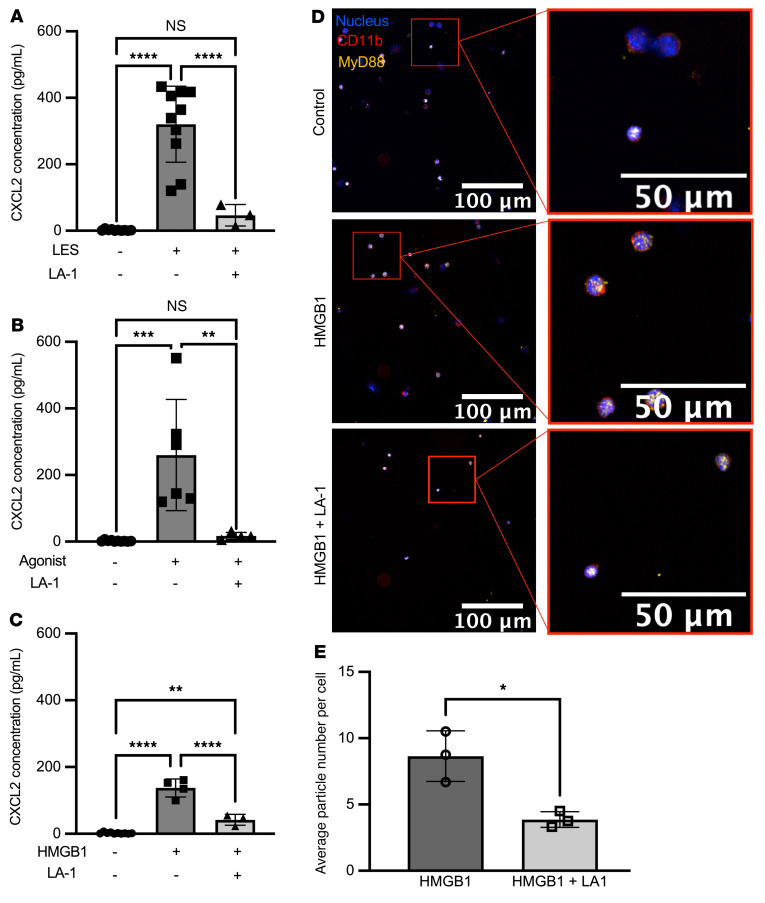
LA-1 decreases CXCL2 production in NCMs. (**A**–**C**) Splenic NCMs were flow cytometry sorted from wild-type (C57BL/6J) mice and then treated with leukadherin-1 (LA-1) (20 μM) 15 minutes prior to the indicated TLR agonists, and CXCL2 was measured in the supernatant 4 hours later. PAM3CSK4, LPS, lysed endothelial supernatant (LES), and HMGB1 were administered at a dose of 10 μg/mL. **P = 0.0019 (**B**); *P* = 0.0020 (**C**); ***P = 0.0001; ****P < 0.0001 by 1-way ANOVA with Tukey’s multiple comparisons test. NS, not significant. (**D**) Splenic NCMs from *Nr4a1-EGFP* mice were flow cytometry sorted and treated with LA-1 (20 μM) followed 15 minutes later by HMGB1 (10 μg/mL), and 4 hours later cytospins of these cells were stained for immunofluorescence analysis (blue: nuclear, green: CD11b, magenta: MyD88). Original magnification, ×400. (**E**) MyD88 particle count in isolated NCMs stimulated with HMGB1 with and without LA-1 treatment. *P = 0.0143 by 2-tailed, unpaired *t* test. Each symbol represents 50,000 NCMs, and approximately 100,000–200,000 NCMs were isolated from an individual mouse.

**Figure 8:**
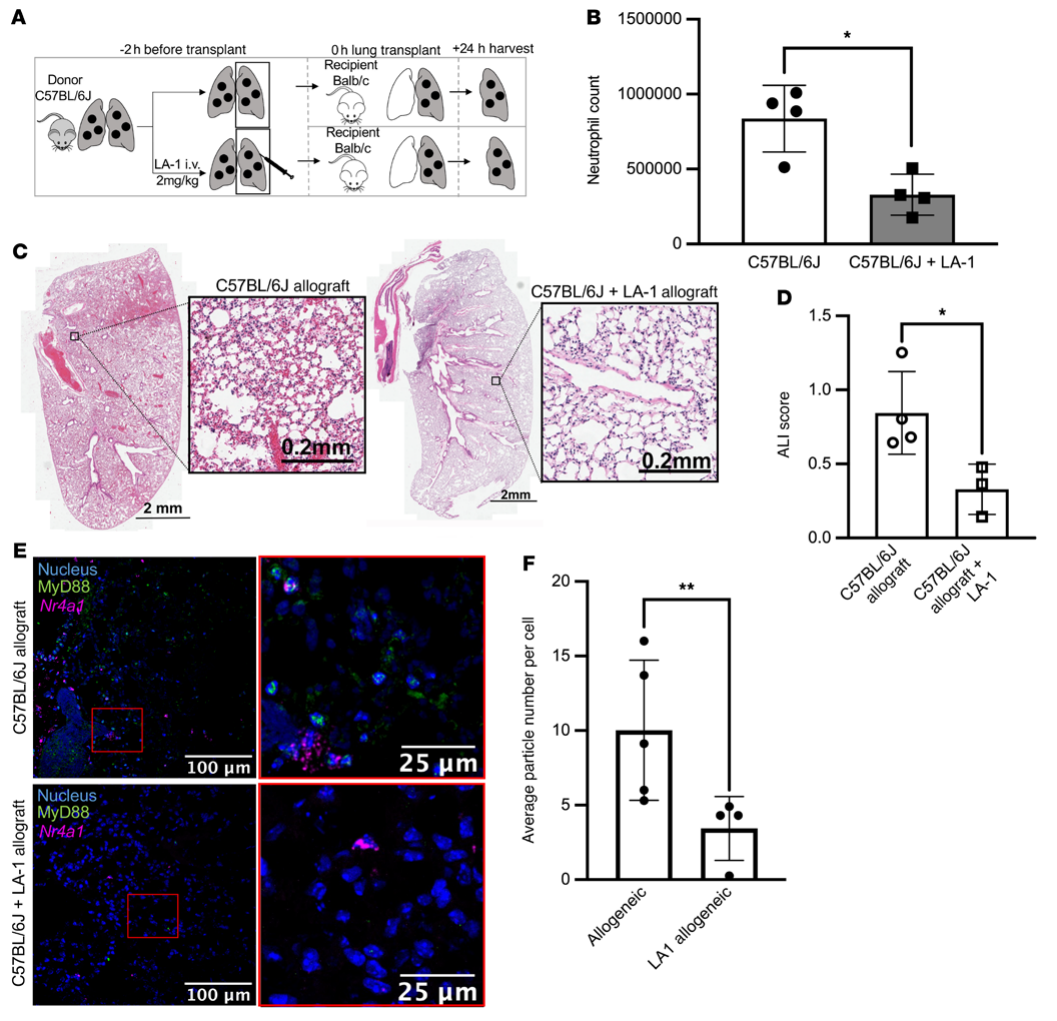
LA-1 prevents PGD in mice. (**A**) Schematic for the allogeneic transplantations. Donor mice were C57BL/6J and recipients were BALB/c. LA-1 was administered (2 mg/kg intravenously) only to the donor. (**B**) Lung allografts were harvested 24 hours after lung transplantation and neutrophil numbers were quantified from lung homogenates using flow cytometry. *P = 0.0128 by 1-way ANOVA with Dunnett’s correction for multiple comparisons, as detailed in Supplemental Figure 5. (**C**) Representative H&E staining of allografts with and without glycyrrhizin treatment. (**D**) Acute lung injury (ALI) scores for the images in **C**. *P = 0.0122 by 1-way ANOVA with Dunnett’s correction for multiple comparisons, as detailed in Supplemental Figure 5. (**E**) Lung sections from allografts of mice with and without glycyrrhizin treatment using RNAscope and immunohistochemical staining (blue: nuclear stain, green: MyD88, magenta: *Nr4a1*). Original magnification, ×400. (**F**) MyD88 particle counts per *Nr4a1*-positive NCM in allografts with and without LA-1 treatment. **P = 0.0070 by 1-way ANOVA with Dunnett’s multiple comparisons test, as detailed in Supplemental Figure 5. Each symbol represents an individual mouse. The controls for ALI score and average particle number per cell are identical to those in Figures 4 and 6.

We next sought to determine whether activation of CD11b is sufficient to reduce CXCL2 release from NCMs and attenuate PGD after lung transplantation. We isolated splenic NCMs from wild-type mice and treated them with LA-1, a CD11b agonist, before stimulation with TLR agonists. NCMs treated with LA-1 showed levels of CXCL2 production similar to untreated cells 4 hours after stimulation with either TLR agonists, LES, or HMGB1 (Figure 7, A–C). Consistently, in flow cytometry–sorted splenic NCMs from *Nr4a1-EGFP* mice, the number of MyD88-containing aggregates induced by HMGB1 was reduced when the cells were pretreated with LA-1 (Figure 7, D and E). To determine whether LA-1 reduced the severity of PGD after lung transplantation, we used an allogeneic model in which C57BL/6J donor lungs were transplanted into BALB/c recipient mice. The donors were pretreated with intravenous LA-1 two hours prior to lung procurement, while the recipient received no treatment (Figure 8A). At 24 hours, allografts treated with LA-1 before procurement had significantly fewer infiltrating neutrophils and reduced histologic evidence of PGD (Figure 8, B–D). Consistent with these findings, the number of MyD88-containing aggregates in NCMs was significantly lower in lung allografts that had been treated with LA-1 prior to lung transplantation (Figure 8, E and F).

## Discussion

In a murine model of lung transplantation, we show that TLR2 and TLR4 are redundantly activated in NCMs retained in the donor lung despite reperfusion to induce the MyD88-mediated release of CXCL2 and the recruitment of recipient neutrophils to the allograft. These recruited neutrophils damage the alveolar capillary barrier, causing lung injury and arterial hypoxemia, characteristic features of human PGD. We discovered an unexpected role for the integrin CD11b in NCMs in lung transplantation–induced injury, which acts as a molecular brake on inflammation, suppressing TLR2- and TLR4-induced CXCL2 release in donor NCMs to reduce PGD severity. We identified HMGB1, a DAMP released from necrotic cells, as a ligand that activates TLR4 in NCMs to initiate PGD. Our findings suggest treatment of donor lungs and recipients with inhibitors of HMGB1 or TLR2/4 as possible therapy for PGD. More importantly, our findings suggest that activators of CD11b, for example LA-1, might be administered to the donor lung alone to reduce the incidence or severity of PGD in patients undergoing lung transplantation while mitigating toxicity in the recipient.

CD11b is a structural protein expressed on the surface of many immune cells. It is necessary for immune cell adherence and movement through the tissue via its interactions with surface proteins on other cells, for example ICAM-1. Our findings extend work from others who identified an inhibitory role for CD11b in the activation of NF-κB and interferon signaling in tissue macrophages (46–52). Specifically, we identify CD11b as a negative regulator of TLR2 and TLR4 signaling through MyD88 in NCMs. The physiologic importance of this regulation is demonstrated by worsening of lung injury when CD11b-deficient lungs were used as donors for syngeneic lung transplantation. Furthermore, the administration of the CD11b agonist LA-1 to the donor lung prior to allogeneic lung transplantation was sufficient to protect against PGD.

To our knowledge, a role for CD11b in attenuating MyD88 signaling in NCMs has not been previously described. In bone marrow–derived and peripheral blood monocyte–derived macrophages, CD11b has been shown to inhibit TLR activation through a process referred to as inside-out signaling (46, 51). At baseline, CD11b is in an inactive, bent conformation. Upon TLR stimulation, MyD88 and other adaptors such as TRIF activate CD11b via phosphoinositide 3-kinase (PI3K) and Rap activation (53). CD11b in its active conformation can activate SYK, which phosphorylates MyD88 and TRIF at specific residues (35, 54, 55). This allows ubiquitination of MyD88 and TRIF by Cbl-b, a single-protein RING-type E3 ligase, targeting them for degradation via the ubiquitin proteasome system. Reduced levels of MyD88 and TRIF attenuate both the NF-κB pathway and the interferon pathways (56, 57). In addition, CD11b has been suggested to inhibit NF-κB signaling by disrupting intracellular trafficking of TLRs or adaptor proteins to the cell surface by suppressing the activation of PI3K and activating calcineurin signaling (52, 54, 58). While NCMs are not macrophages, they share many features, including adherence to the vascular wall, crawling against the flow of blood, and efferocytosis of apoptotic endothelial cells (59). Hence, we speculate that the mechanisms by which CD11b and MyD88 interact in NCMs are similar to those described above in bone marrow–derived and peripheral blood monocyte–derived macrophages from mice and humans, respectively. Unfortunately, the small number of NCMs in mice and humans makes confirmation of these mechanisms challenging.

In the 5 years before the COVID-19 pandemic, the number of lung transplants performed yearly in the United States had increased by over 31% (60). We were the first to report the feasibility of lung transplantation for the treatment of patients with end-stage fibrosis secondary to COVID-19, and now several centers internationally offer this procedure. Historically, the incidence of PGD is between 10% and 25% (60). In our cohort of 35 patients who underwent lung transplantation for COVID-19, the cumulative incidence of PGD (grades 1–3), recorded 72 hours after transplantation, was dramatically higher (70% compared with a rate of 20.8%). While 95% of our cohort of COVID-19 patients are alive at the time of this writing, the reported all-cause 30-day mortality in lung transplant recipients who develop PGD is 42.1% versus 6.1% in those without, and 64.9% at 1 year versus 20.4% in those without (61). More importantly, PGD remains an important risk factor for the development of chronic lung allograft dysfunction, the major cause of late mortality in lung transplant recipients (62). The expanding pool of patients undergoing lung transplantation for COVID-19 and the high rates of PGD in this population highlights the need for targeted therapies to prevent PGD.

Our findings have potential implications for therapy. To expand organ availability, many centers preserve lungs using ex vivo lung perfusion (EVLP), in which a donor lung is ventilated and perfused in a temperature-controlled closed system. In addition to prolonging graft viability, EVLP offers the opportunity to treat the donor lung prior to implantation (63–65). Our data suggest several complementary approaches that could be used to treat donor lungs in an EVLP system prior to implantation, including LA-1 or similar CD11b agonists and small molecule inhibitors of HMGB1, including glycyrrhizin. Our studies using LA-1 are particularly interesting, as the drug was effective when administered to the donor lung but not the recipient, reducing the chances of off-target effects in the recipient. In contrast, strategies to inhibit signaling through individual TLRs are unlikely to be effective, as these pathways are redundant.

This study presents some limitations. While we have shown that CD11b plays a role in the development of PGD in murine lung transplantation, we still do not understand the role of CD11b in human lung transplantation. Second, we selected a panel of DAMPs based on their reported ability to activate TLR2 and TLR4, which were implicated in our genetic experiments. It is likely that there are other DAMPs beyond HMGB1 that may signal through these receptors. Indeed, while others have reported that HMGB1 signals through TLR2, our in vitro data suggest HMGB1 primarily signals through TLR4 in NCMs, leaving open the question of how TLR2 is activated after lung transplantation. Third, we observed MyD88 aggregates in multiple cell populations after lung transplantation; hence, these findings are insufficient alone to causally implicate activation of MyD88 in NCMs in neutrophil recruitment and PGD. Instead, this conclusion is supported by our data showing protection against PGD in mice with a targeted knockout of MyD88 in NCMs and our previously published work, which has since been confirmed by others (24, 66–68). Lastly, there is a lack of long-term murine studies to determine whether the use of LA-1 or other therapies that reduce PGD will improve long-term outcomes, including chronic lung allograft dysfunction.

In conclusion, we show that retained donor NCMs in lung allografts lead to PGD through redundant activation of TLR2 and TLR4 in response to HMGB1. Loss of CD11b, a negative regulator of TLR signaling through MyD88, increases CXCL2 production and worsens PGD. Administration of a CD11b agonist, LA-1, to the donor lung effectively prevents PGD. Our results elucidate a specific mechanism driving PGD, with implications for clinical therapy targeting the donor lung prior to lung transplantation.

## Methods

### Human patients

Lung biopsies obtained for the above studies were obtained from lung allografts treated using standard perfusion protocols prior to implantation. A detailed description of these protocols can be found in the methods of Zheng et al. (24). Briefly, lungs were procured and maintained in a low-potassium dextran (Perfadex) solution cooled on ice. Biopsies were obtained prior to procurement, after immediate removal from the cooled Perfadex solution, and 90 minutes following reperfusion.

### Mice and procedures

The genotype of all animal strains was confirmed by sequencing of ear punches by a third-party vendor (Transnetyx). C57BL/6J, BALB/cJ, B6.129S4-*Itgam^tm1Myd^*/J (*Itgam^–/–^*), C.129(B6)-*Tlr2^tm1Kir^*/J (*Tlr2^–/–^*), B6N.129S1-*Tlr3^tm1Flv^*/J (*Tlr3^–/–^*), B6(Cg)-*Tlr4^tm1.2Karp^*/J (*Tlr4^–/–^*), B6.129S1-*Tlr7^tm1Flv^*/J (*Tlr7^–/–^*), C57BL/6J-*Tlr9^em1.1Ldm^*/J (*Tlr9^–/–^*), B6N.B6-Tg(Nr4a1-EGFP/cre)820Khog/J, and B6*.*129P2(SJL)-*Myd88^tm1Defr^*/J mice were purchased through The Jackson Laboratory. The mouse strain *Nr4a1-EGFP/cre* *Myd88^fl/fl^* was produced by crossbreeding B6N.B6-Tg(Nr4a1-EGFP/cre)820Khog/J mice and B6.129P2(SJL)-*Myd88^tm1Defr^*/J mice for the final offspring with *Nr4a1* hemizygotes with homozygous knockout of *Myd88*. *Tlr2^–/–^* *Tlr4^–/–^* mice were produced by crossbreeding *Tlr2^–/–^* with *Tlr4^–/–^* mice, with final offspring being homozygous knockouts for both *Tlr2* and *Tlr4*. All mice were housed in the Center for Comparative Medicine at Northwestern University. Mice used in the following procedures were aged 10–16 weeks and between 24 and 28 g body weight.

#### Orthotopic vascularized lung transplantation.

Orthotopic vascularized left lung transplantation was performed in accordance with previously described protocols (24). In brief, donor mice were anesthetized using xylazine (10 mg/kg) and ketamine (100 mg/kg) before intubation and were re-dosed as needed to maintain deep anesthesia during the procedure. Ventilation was maintained on room air. Thoracotomy was performed and the donor lung was flushed using 3 mL of sterile saline solution. The heart-lung block was excised and kept at 4°C in preservative solution. The donor lung was then prepared for anastomosis in the recipient mouse by applying custom cuffs made from Teflon intravenous catheters to the pulmonary artery, vein, and bronchus and secured with a 10-0 ligature. The graft was stored at 4°C for 90 to 120 minutes for ischemic time. Recipient mice were prepared for surgery by receiving subcutaneous buprenorphine (0.1 mg/kg) 30 minutes prior to surgery start time and as needed following the procedure every 6 hours. After the recipient mice were intubated, a left-sided thoracotomy was performed at the third intercostal space. The recipient left lung was clamped and removed from the thoracic cavity to allow for dissection of the recipient pulmonary artery, vein, and bronchus. The pulmonary artery and vein were occluded using 8-0 nylon ligatures. The donor left lung was implanted by anastomosing each cuff to the respective native counterpart with 10-0 ligatures. The ligatures occluding the vein and then the artery were removed. The chest wall and incision were closed and the recipient was weaned off the ventilator when spontaneous respirations resumed. No antibiotics or immunosuppression were used.

Control allogeneic murine lung transplants, C57BL/6J to BALB/c, were performed over several days, where *n* = 1–2 were performed per day. Controls were divided contemporaneously between Figures 3, 6, and 8.

#### Glycyrrhizin injection.

Glycyrrhizin (Sigma-Aldrich, PHR1516) was used at 4 mg/kg body weight, diluted in 50 μL of phosphate-buffered saline (PBS) and instilled retro-orbitally to both the donor mouse 30 minutes prior to lung procurement and to the recipient mouse immediately following reperfusion of the lung.

#### LA-1 injection.

LA-1 (Sigma-Aldrich, SML0886) dissolved in PBS was used at 6 mg/kg body weight diluted in 50 μL of PBS. The solution was gently made into a solution by gentle heat at 37°C with vortexing. The injection was instilled retro-orbitally to the donor mouse 2 hours prior to lung procurement.

#### Arterial blood gases.

Recipient mice 24 hours after transplantation were anesthetized using subcutaneous buprenorphine (0.1 mg/kg) and intubated. A thoracotomy was performed and mice were given subcutaneous heparin. The right-side hilum (native) was exposed where the pulmonary artery, vein, and bronchus was clamped using a microvessel clamp for 3 minutes. At 3 minutes, an arterial blood sample was collected intracardially and promptly analyzed using the Abbott iStat arterial blood gas machine.

### Isolation of NCMs

Spleens were passed through a 40 μm strainer to form a single-cell suspension. After red blood cell lysis, the spleens were depleted of CD3^+^ and CD19^+^ cells using magnetic beads (Miltenyi Biotec, 130-094-973 and 130-121-301, respectively) and depletion (LD) columns. The remaining cells were stained using an antibody cocktail found in Supplemental Table 1 for 30 minutes. NCMs were sorted using a BD FACSMelody sorter at the Northwestern University Robert H. Lurie Comprehensive Cancer Center Flow Cytometry Core Facility. A representative gating strategy can be found in Supplemental Figure 1.

### Cell culture

Splenic NCMs obtained from flow cytometry were plated at 50,000 cells in 100 μL of RPMI 1640 supplemented with 10% fetal bovine serum (FBS) and 1% penicillin-streptomycin in CellStar cell-repellent 96-well plates. After experimentation, cells and media were collected and pelleted. The supernatant was carefully put into a separate bullet tube and stored at –30°C until it was used for protein analysis.

### In vitro reagents

The following agonists were used for in vitro studies: PAM3CSK4 (Sigma-Aldrich, 506350), LPS (Sigma-Aldrich, *Escherichia coli* O111:B4, L2630), polyinosinic-polycytidylic acid [poly(I:C)] sodium salt (Sigma-Aldrich, P1530), resiquimod (Sigma-Aldrich, SML0196), ODN 1826 (InvivoGen, tlrl-1826), and HMGB1 Protein, Mouse Recombinant (SinoBiological, 50913-M01H). All agonists were dosed at 1 μg. LA-1 and glycyrrhizin mentioned above were used at 20 μM concentration 30 minutes before agonists were added.

### Cell fractionation

LES was made from fractionated cultured lung endothelial cells. Primary human lung endothelial cells were cultured to confluence in complete RPMI 1640 (with 10% FBS and 1% penicillin-streptomycin). Cells were then scraped, pelleted, and resuspended in 500 μL of fractionation buffer (10 mM Tris-HCl pH 7.5, 1 mM EDTA, and 10 μL/mL TPCK, PMSF, and leupeptide). A motorized pestle was used in the suspension for 2 rounds of 1 minute each.

### Multiplex ELISA

Cytokine levels expressed from NCMs were measured using a custom multiplex ELISA plate (MSD) with group 1 mouse biomarkers. Assays were performed according to MSD’s protocol.

### HMGB1 ELISA

Blood samples were collected from lung transplant recipient mice 2 hours after transplantation in polystyrene bullet tubes. Peripheral venous blood samples were collected from patients immediately prior to the transplantation and 24 hours after reperfusion. Sera from the samples were collected by allowing the blood sample to clot for 30 minutes and then centrifuged for 10 minutes at 1,500*g*. The serum was saved at –30°C until use. HMGB1 levels were analyzed using an HMGB1 Express ELISA (Tecan Bio).

### Murine lung isolation and multicolor flow cytometry

Lungs harvested 24 hours following orthotopic lung transplantation were processed to a single-cell suspension and were stained according to a previously described method (69). Antibodies used for lung staining can be found in Supplemental Table 1. Samples were run on a BD FACSymphony A5-Laser Analyzer at the Northwestern University Robert H. Lurie Comprehensive Cancer Center Flow Cytometry Core Facility and then subsequently analyzed with FlowJo v10.6.2 (FlowJo).

### Histology and H&E staining

For histological analyses, lungs to be harvested were gently flushed intracardially with 10 mL of cold Hank’s balanced salt solution (HBSS). The entire heart-lung block was removed from the thoracic cavity with ample length of trachea intact. The trachea was cannulated with a custom cut Teflon venous catheter and secured with a ligature. A 10 mL solution of 10% formalin was instilled via the catheter at a pressure of 10 cmH_2_O and then fixed in 10% formalin for 24 to 48 hours. The lungs were subsequently embedded in paraffin. Whole lungs were serially sectioned and stained with H&E. Images were acquired using a Nikon TE-2000 U light microscope. Acute lung injury was quantified as per recommendation as described in Matute-Bello et al. (70).

### RNAScope and immunohistochemistry

The RNAScope procedure was performed using an optimized protocol based on standard assay protocols from ACDBio. Briefly, paraffin-embedded samples were serially sectioned at 5 μm slices on charged slides. Slides were deparaffinized in 100% xylene for 5 minutes, twice. The slides were then rehydrated in 100% ethanol for 2 minutes each, twice. Endogenous peroxidase activity was blocked using hydrogen peroxide for 10 minutes followed by 2 washes in water. Antigen retrieval was performed using 1× target retrieval buffer between 95°C and 100°C for 15 minutes followed by 15 seconds in water and 3 minutes in 100% ethanol. Digestion for RNAScope was done using mild digestion times with protease III and the probe was added to the slides (RNAscope Probe- Mm- NR4a1, 322335). Amplification and staining of the probe were done per standard protocol. The slides were then washed in 1× Tris-buffered saline (TBS) with 0.05% Tween (TBST) for 5 minutes, twice. Slides were then blocked using 10% normal goat serum in TBS with 1% bovine serum albumin (BSA) for 30 minutes at room temperature. Blocking solution was flicked off and the primary antibody (anti-MyD88) in TBS with 1% BSA was applied for 60 minutes at room temperature in a dark humidified chamber. Slides were washed twice using TBST for 5 minutes each. The secondary antibody (donkey anti–rabbit IgG, Alexa Fluor 488) diluted in TBS with 1% BSA was applied for 30 minutes at room temperature in a dark, humidified chamber and subsequently washed in TBST twice for 5 minutes each. RNAscope DAPI solution was applied to the slides for 30 seconds. Solution was flicked off and Anti-fade Gold Mountant was applied and a coverslip was placed on top. Slides were cured overnight. Images were taken at the Northwestern University Center for Advanced Microscopy using a Nikon W-1 Spinning Disk Confocal microscope. Final images were rendered via Fiji (NIH). Antibodies and concentrations used can be found in Supplemental Table 1.

### Immunofluorescence imaging

NCMs in cell culture were isolated onto microscopy slides using a Cytospin for 5 minutes at 113*g* at medium acceleration. Slides were washed twice using 1× PBS. Slides were then fixed used 4% paraformaldehyde for 20 minutes and then washed twice. Slides were then permeabilized using 1× PBS with 1% Triton X-100 for 20 minutes and then washed. Slides were then blocked using 10% normal goat serum in PBS with 1% BSA for 30 minutes at room temperature. Blocking solution was flicked off and the primary antibody (anti-MyD88, anti-CD11b) in PBS with 1% BSA was applied for 60 minutes at room temperature in a dark humidified chamber. Slides were washed twice using TBST for 5 minutes each. The secondary antibody (donkey anti–rabbit IgG, Alexa Fluor 647) diluted in PBS with 1% BSA was applied for 30 minutes at room temperature in a dark, humidified chamber and subsequently washed in PBST twice. Slides were counterstained using a 1:1000 dilution of Hoechst 33342 for 5 minutes in the dark. Solution was flicked off and Prolong Gold AntiFade Mountant was applied and a coverslip was placed on top. Slides were cured overnight. Images were taken at the Northwestern University Center for Advanced Microscopy using a Nikon W-1 Spinning Disk Confocal microscope. Final images were rendered via Fiji. Antibodies and concentrations used can be found in Supplemental Table 1.

### Statistics

Data analysis was performed using Prism 9 (GraphPad Software). Results are shown as mean ± SD. The statistical significance test performed is provided in the figure legends. A *P* value of less than 0.05 was considered significant. For the primary endpoint of neutrophil recruitment to the allograft after transplantation, experimental groups were organized into cohorts as presented in the figures, but because the surgeon could perform only 5 to 7 transplants per week, the experiments are not truly contemporaneous. Hence, we compared controls with interventions using an ANOVA with a Dunnett’s test to correct for multiple comparisons (Supplemental Figure 5). It is not possible to obtain histologic sections and measure neutrophil recruitment in lung homogenates simultaneously. Hence, the acute lung injury scores and particle counts in Figures 4, 6, and 8 were performed using the same controls as noted in the figure legends. Statistical comparisons were performed with an ANOVA followed by a Dunnett’s correction for multiple comparisons (Supplemental Figure 5).

### Study approval

All procedures were approved by The Institutional Animal Care and Use Committee (IS00002248) at Northwestern University. All animals received humane and ethical care in compliance with the NIH *Guide for the Care and Use of Laboratory Animals* (National Academies Press, 2011). The guidelines for laboratory animal care was formulated by the National Society for Medical Research. The human research protocol was approved by the Northwestern University Institutional Review Board (STU00106589 and STU00201397). All study subjects were informed and provided written consent prior to study.

## Author contributions

MQ contributed to conceptualization, study design and methodology, experimentation, data collection and formal analysis, and manuscript writing. SC, QW, HS, SR, MA, MK, and DK conducted experiments. EL and AVM contributed to methodology, validation, formal analysis, and visualization. GRSB and AB contributed to conceptualization, methodology, validation, formal analysis, investigation, resources, data curation, writing, visualization, supervision, project administration, and funding acquisition.

## Supplementary Material

Supplemental data

## Version 1 07/15/2022

Electronic publication
