# Supplementary material for: CD11b suppresses TLR activation of nonclassical monocytes to reduce primary graft dysfunction after lung transplantation
Source: J Clin Invest. 2022 Jul 15;132(14):e157262. doi: 10.1172/JCI157262 (PMC9282933; doi:10.1172/JCI157262)
Supplement: Supplemental data [file jci-132-157262-s029.pdf]

SUPPLEMENTAL MATERIAL

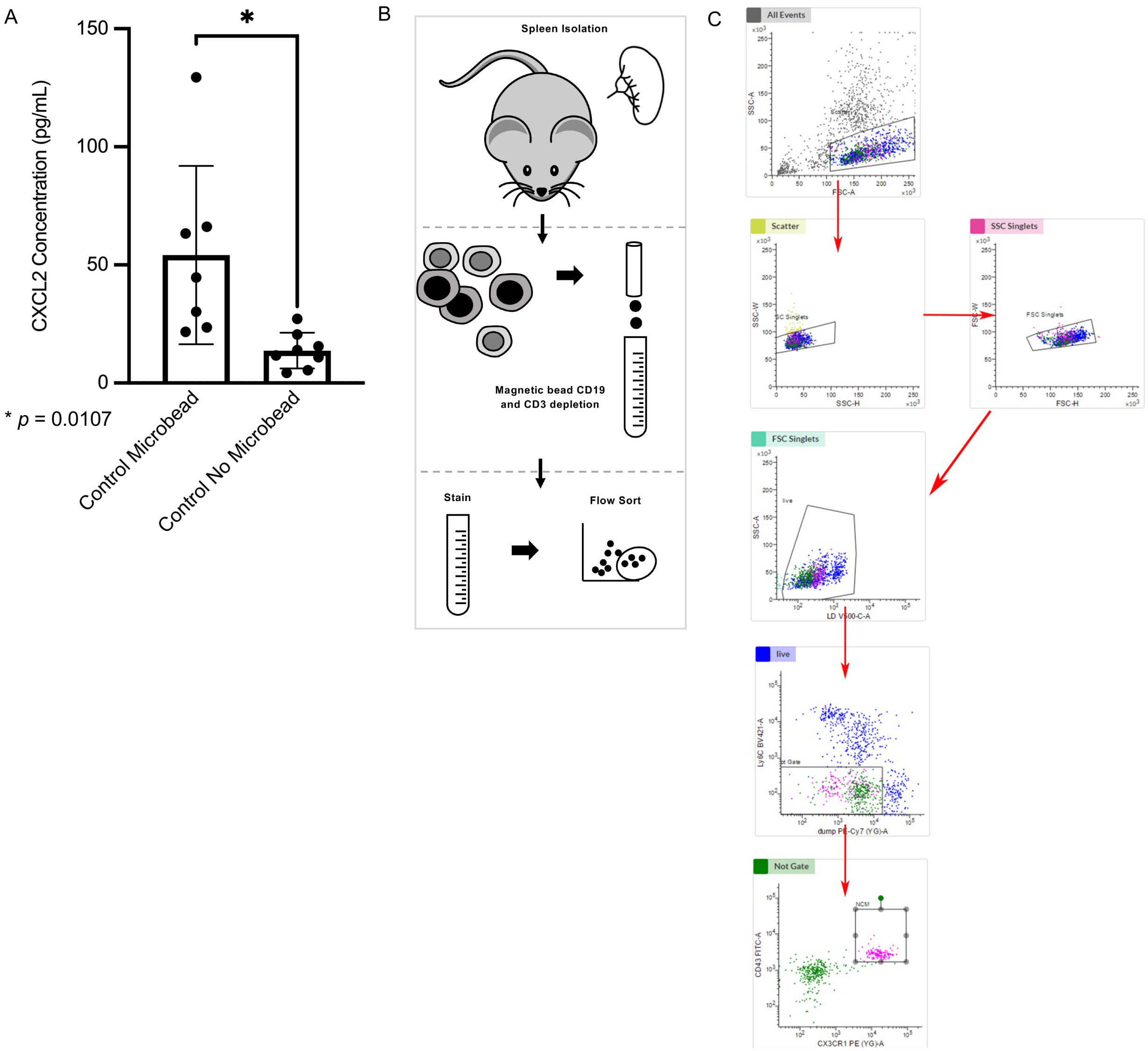

Supplemental Figure 1. NCM negative selection flow cytometry sorting strategy.
(**S1A**) Splenic non-classical monocytes isolated with and without CD11b microbeads in the absence of stimulation. *P*-value was calculatd using an unpaired t-test. (**S1B**) Splenic non-classical monocytes were isolated using a negative selection strategy where CD19+ and CD3+ cells are depleted from single cell suspensions using magnetic beads. (**S1C**) The remaining cell suspension is flow cytometry sorted for non-classical monocytes by negatively selecting cells without CD19, CD3, MHCII, MerTK, Ly6G, NK1.1, and CD11c and then positively selecting CD43+CX3CR1^{hi} cells.

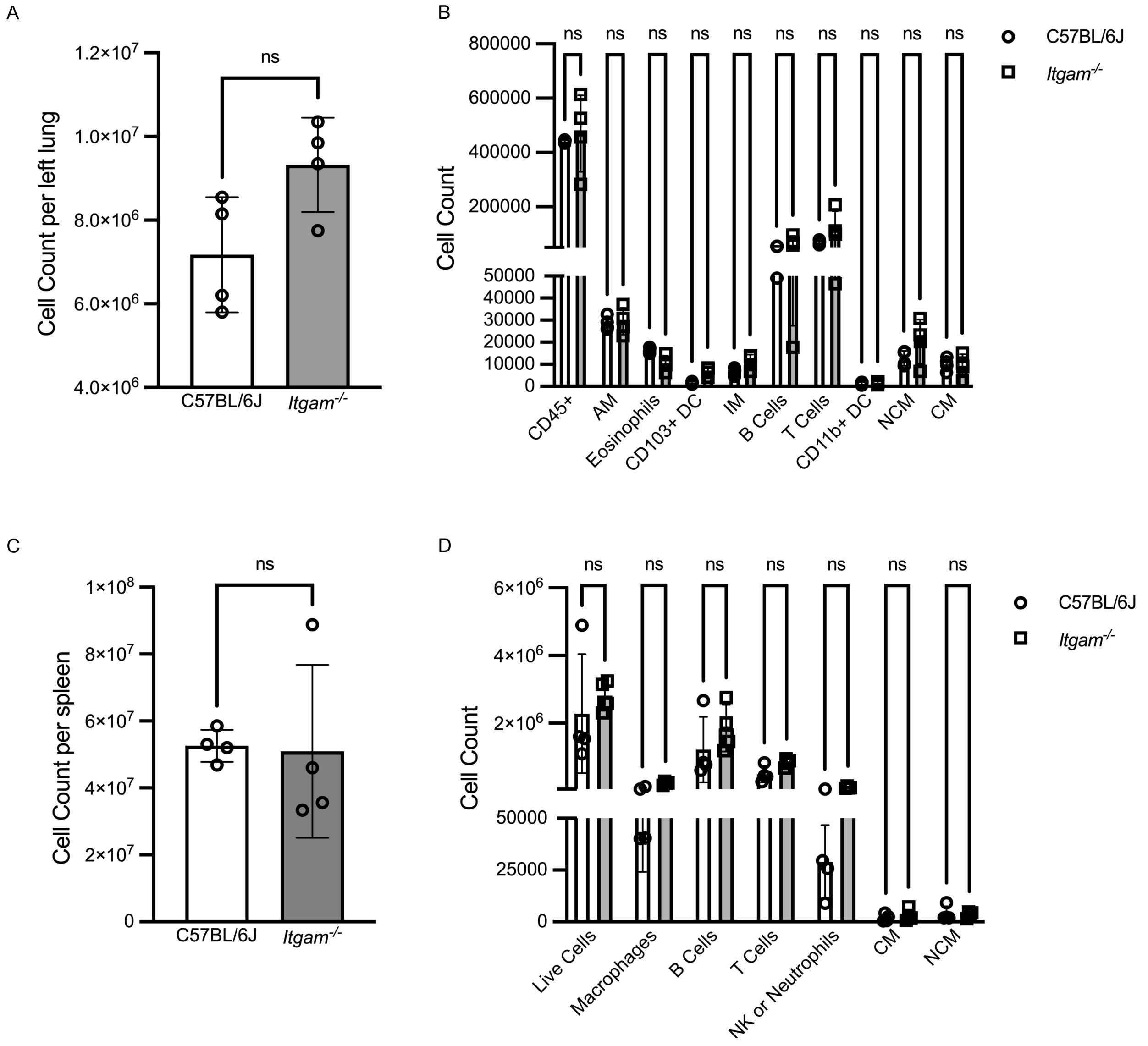

Supplemental Figure S2. *Itgam*$^{-/-}$ mice spleen and lung phenotypes are similar to C57BL/6J controls at baseline
(**S2A**) Cell count per left lung in C57BL/6J and *Itgam*$^{-/-}$ mice. *P*-value was calculated using an unpaired t-test. (**S2B**) Immune cell counts in C57BL/6J and *Itgam*$^{-/-}$ lungs. *P*-values were calculated using a one-way ANOVA. (**S2C**) Cell count in spleens of C57BL/6J and *Itgam*$^{-/-}$ mice. *P*-value were calculated using an unpaired t-test. (**S2D**) Immune cell counts in C57BL/6J and *Itgam*$^{-/-}$ spleens. *P*-values were calculated using a one-way ANOVA. Each point represents an individual mouse.

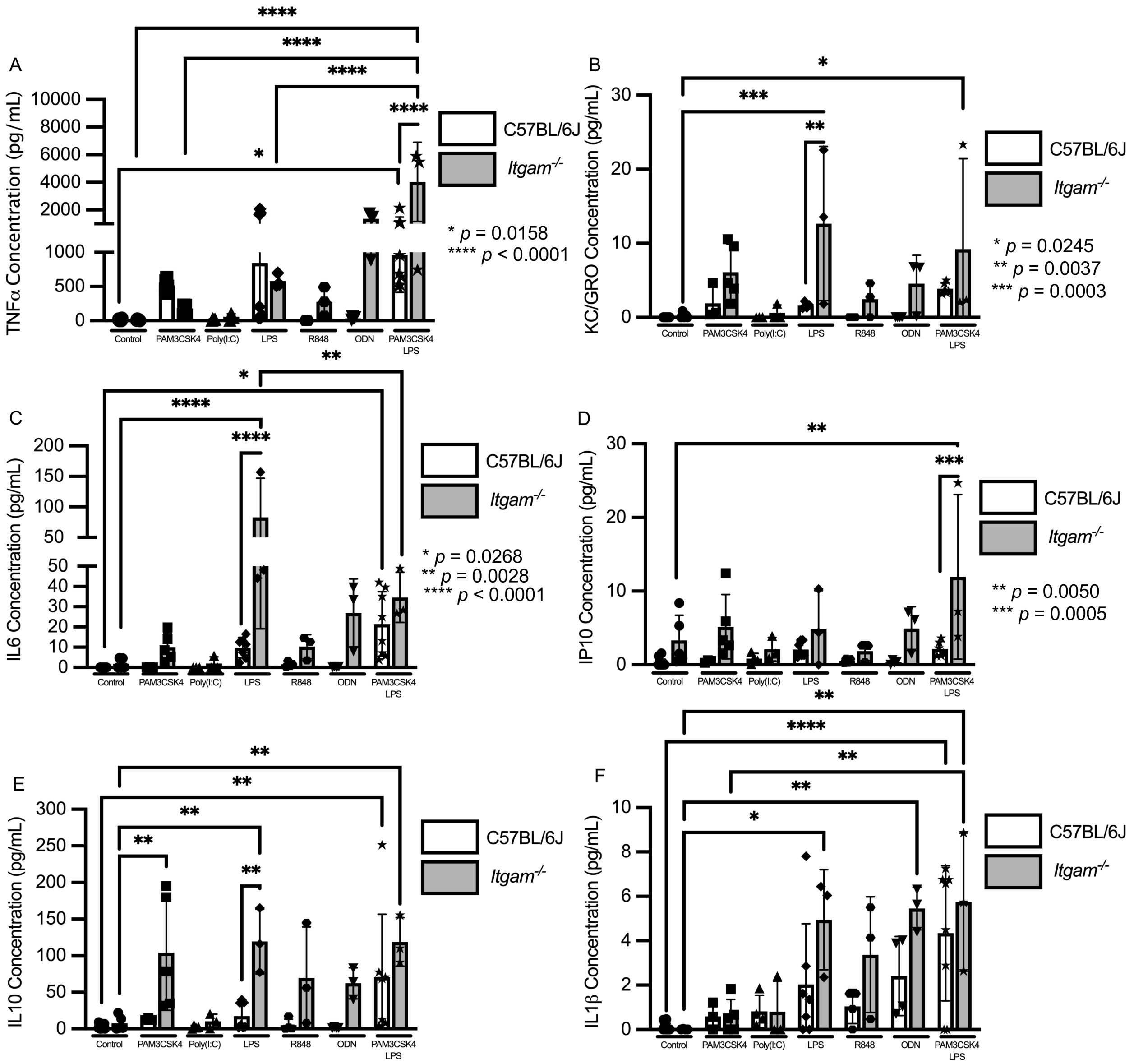

C57BL/6J Control:C57BL/6J PAM3CSK4/LPS ** $p = 0.0075$
Itgam$^{-/-}$ Control: *Itgam*$^{-/-}$ PAM3CSK4 ** $p = 0.0066$
Itgam$^{-/-}$ Control: *Itgam*$^{-/-}$ LPS ** $p = 0.0063$
Itgam$^{-/-}$ Control: *Itgam*$^{-/-}$ PAM3CSK4/LPS ** $p = 0.0068$
C57BL/6J LPS: *Itgam*$^{-/-}$ LPS ** $p = 0.0098$

C57BL/6J Control:C57BL/6J PAM3CSK4/LPS **** $p < 0.0001$
Itgam$^{-/-}$ Control: *Itgam*$^{-/-}$ LPS * $p = 0.0140$
Itgam$^{-/-}$ Control: *Itgam*$^{-/-}$ ODN ** $p = 0.0042$
Itgam$^{-/-}$ Control: *Itgam*$^{-/-}$ PAM3CSK4/LPS ** $p = 0.0020$
Itgam$^{-/-}$ PAM3CSK4:*Itgam*$^{-/-}$ PAM3CSK4/LPS ** $p = 0.0049$

Supplemental Figure S3. *Itgam*$^{-/-}$ increases production in multiple inflammatory cytokines in response to TLR2 and TLR4 agonists. Splenic non-classical monocytes were flow cytometry sorted from wildtype (C57BL/6J) and *Itgam*$^{-/-}$ mice and stimulated with TLR agonists as indicated for 4 hours after which the supernatants are collected for ELISA analysis: TNFα (**S3A**), KC/GRO (**S3B**), IP10 (**S3C**), IL10 (**S3D**), IL6 (**S3E**), and IL1β (**S3F**). *P*-values were calculated by a one-way ANOVA with Sidak's multiple comparisons. PAM3CSK4, polyinosinic:polycytidylic acid (Poly(I:C)), lipopolysaccharide (LPS), Resiquimod (R848), and oligodeoxynucleotide (ODN), and PAM3CSK4 and LPS. All agonists were administered at a dose of 1μg. Each symbol represents 50,000 nonclassical monocytes, ~100,000 – 200,000 cells are isolated in an individual mouse.

A

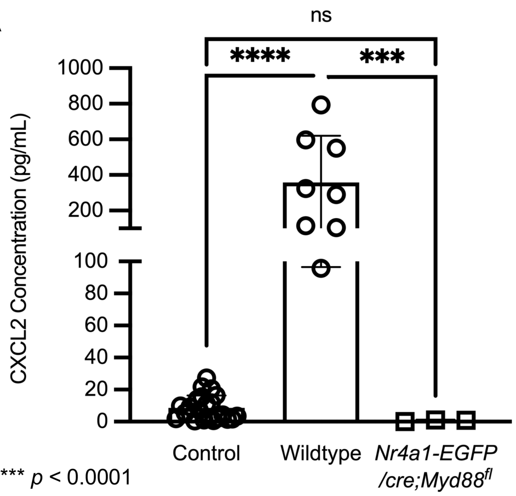

**** $p < 0.0001$

Supplemental Figure S4. *Nr4a1-EGFP/cre;Myd88*^{fl} NCM do not respond to TLR2 and TLR4 agonists.
(**S4A**) CXCL2 concentration from splenic non-classical monocytes isolated from C57BL/6J and *Nr4a1-EGFP/cre;Myd88*^{fl} mice stimulated with 1μg of TLR2 (PAM3CSK4) and TLR4 (LPS) for four hours. *P*-values were calculated using a one-way ANOVA.

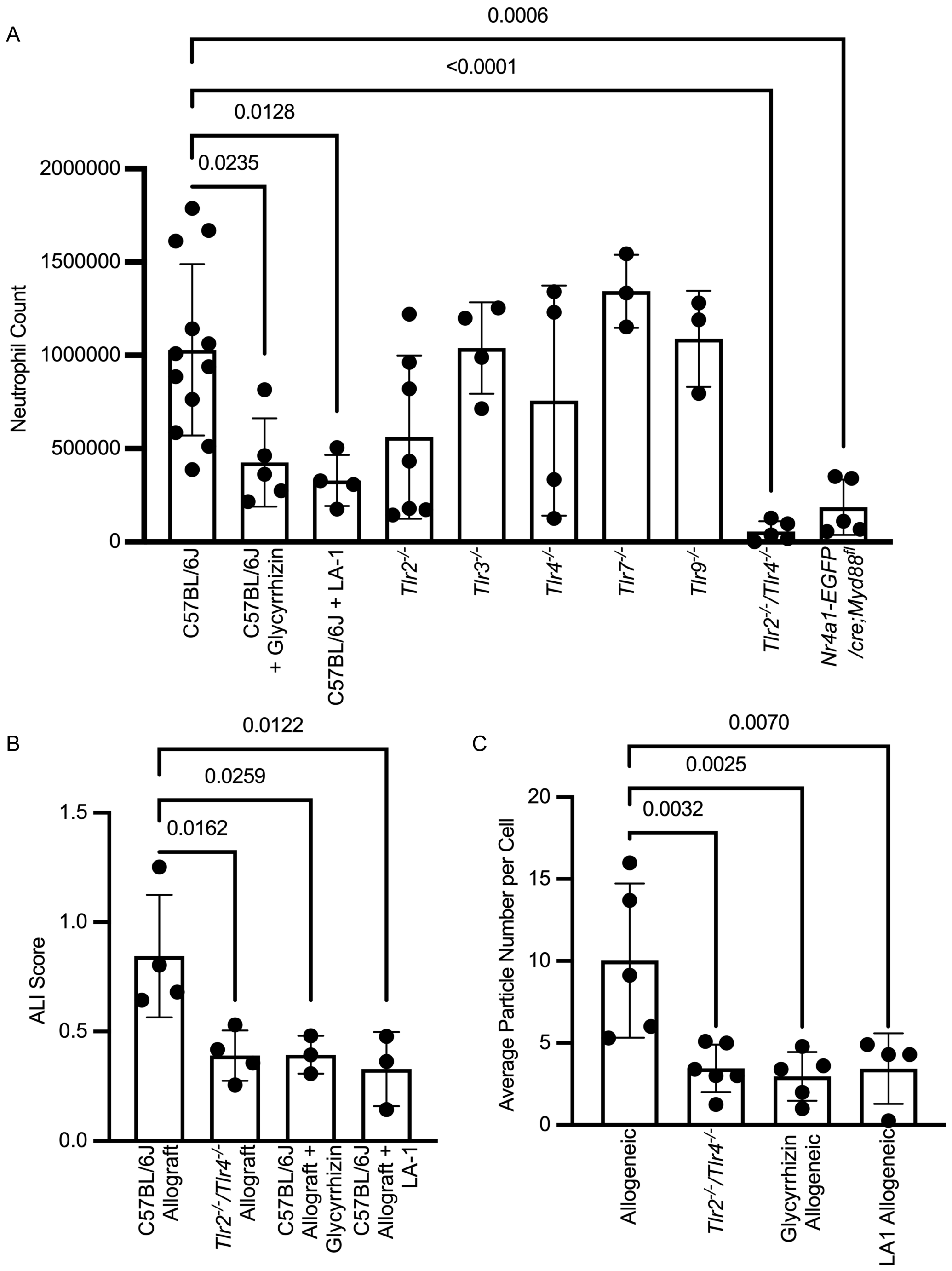

Figure S5. Multivariate statistical analyses found for lung transplant neutrophils, ALI scores, and particle counts
(**S5A**) To minimize variation between experiments, all experiments in this manuscript were performed by the same murine lung transplant surgeon. For the primary endpoint of neutrophil recruitment to the allograft, experiments were conducted in individual cohorts of mice as presented in Figures 3, 6 and 8. As these cohorts could not be truly contemporaneous, comparison between the untreated wild-type control transplants and transplants after different interventions were made with a one way ANOVA followed by a Dunnett's test to correct for multiple comparisons. These results are referenced in the main legends for Figures 3, 6 and 8. (**S5B**) A group of control mice and mice treated with the indicated interventions were used to measure ALI scores. P values were compared using a one-way ANOVA followed by a Dunnett's test for multiple comparisons and are referenced in Figures 3,6 and 8. (**S5C**) The same group of control mice and mice treated with the indicated interventions in Figure S5B were used to measure ALI scores. P values were compared using a one-way ANOVA followed by a Dunnett's test for multiple comparisons and are referenced in Figures 3,6 and 8

Supplemental Table 1. Flow cytometry analysis and sorting antibody cocktails.

Fluorochrome	Antibody	Company/Clone	Dilution
Lung Transplant Myeloid			
eF450	Ly6C	eBioscience HK1.4	1:500
eF506	Fixable Viability	eBioscience	1:500
FITC	CD45	BioLegend 30-F11	1:250
PerCPCy5.5	IA/IE	BioLegend MS/114.15.2	1:1000
PE	CD64	BioLegend X54-517.1	1:500
PECF594	SiglecF	BD Bioscience E50-2440	1:500
PECy7	CD11c	BD Bioscience HL3	1:500
APC	CD24	eBioscience M1/69	1:500
APCCy7	CD11b	BD Bioscience M1/70	1:500
	Ly6G	BD Bioscience IA8	1:250
AF700	NK1.1	BD Bioscience PK136	1:166
No CD11b NCM sort			
BV421	Ly6C	eBioscience HK1.4	1:500
eF506	Fixable Viability	eBioscience	1:500
FITC	CD43	eBioscience eBioR2/60	1:500
PE	CXCR1	BioLegend SA011F11	1:500
	CD11c	BD Bioscience HL3	1:500
	IA/IE	BioLegend M/115.15.2	1:1000
	Ly6G	BioLegend IA8	1:250
	MerTK	eBioscience DS5MMER	1:500
PECy7	NK1.1	Life Technologies PK136	1:166
No CD11b lung phenotype			
BUV496	CD24	BD Bioscience M1/69	1:500
eF450	Ly6C	eBioscience HK1.4	1:500
eF506	Fixable Viability	eBioscience	1:500
FITC	CD3	ThermoFisher 17A2	1:166
PerCPCy5.5	IA/IE	BioLegend MS/114.15.2	1:1000
PE	CX3CR1	BioLegend SA011F11	1:500
PECF594	SiglecF	BD Bioscience E50-2440	1:500
PECy7	CD11c	BD Bioscience HL3	1:500
APC	CD19	BD Bioscience 1D3	1:500
APCCy7	CD45	BD Bioscience 30-F11	6:1000
	Ly6G	BD Bioscience IA8	1:250
AF700	NK1.1	BD Bioscience PK136	1:166
No CD11b spleen phenotype			
eF450	Ly6C	eBioscience HK1.4	1:500
eF506	Fixable Viability	eBioscience	1:500
FITC	CD3	ThermoFisher 17A2	1:166
PerCPCy5.5	IA/IE	BioLegend MS/114.15.2	1:1000
PE	CX3CR1	BioLegend SA011F11	1:500
PECF594	CD19	BD Bioscience 1D3	1:166
PECy7	CD11c	BD Bioscience HL3	1:500
APC	F4/80	eBioscience BM8	1:500
	Ly6G	BD Bioscience IA8	1:250
AF700	NK1.1	BD Bioscience PK136	1:166
Immunohistochemistry/Immunocytochemistry			
Uncong	MyD88	Novus Biologicals	1:200
AF594	CD11b	BioLegend M1/70	1:100
AF488	Donkey Anti-Rabbit IgG	Abcam	1:200
AF647	Donkey Anti-Rabbit IgG	Abcam	1:200
Hoescht 33342	nuclear	Life Technologies	1:1000
